# Conversion of a High-Altitude Temperate Forest for Agriculture Reduced Alpha and Beta Diversity of the Soil Fungal Communities as Revealed by a Metabarcoding Analysis

**DOI:** 10.3389/fmicb.2021.667566

**Published:** 2021-06-21

**Authors:** Yendi E. Navarro-Noya, Nina Montoya-Ciriaco, Ligia C. Muñoz-Arenas, Stephanie Hereira-Pacheco, Arturo Estrada-Torres, Luc Dendooven

**Affiliations:** ^1^Laboratory of Biotic Interactions, Centro de Investigación en Ciencias Biológicas, Universidad Autónoma de Tlaxcala, Tlaxcala, Mexico; ^2^Doctorado en Ciencias Biológicas, Universidad Autónoma de Tlaxcala, Tlaxcala, Mexico; ^3^Facultad de Ingeniería Ambiental, UPAEP, Puebla, Mexico; ^4^Laboratory of Soil Ecology, CINVESTAV-IPN, Ciudad de México, Mexico; ^5^Centro Tlaxcala de Biología de la Conducta, Universidad Autónoma de Tlaxcala, Tlaxcala, Mexico

**Keywords:** arbuscular mycorrhiza, deforestation, ectomycorrhizal fungi, pathotrophs, high mountain forest, land-use change

## Abstract

Land-use change is one of the most important drivers of change in biodiversity. Deforestation for grazing or agriculture has transformed large areas of temperate forest in the central highlands of Mexico, but its impact on soil fungal communities is still largely unknown. In this study, we determined how deforestation of a high-altitude temperate forest for cultivation of maize (*Zea mays* L.) or husbandry altered the taxonomic, phylogenetic, functional, and beta diversity of soil fungal communities using a 18S rRNA metabarcoding analysis. The true taxonomic and phylogenetic diversity at order *q* = 1, i.e., considering frequent operational taxonomic units, decreased significantly in the arable, but not in the pasture soil. The beta diversity decreased in the order forest > pasture > arable soil. The ordination analysis showed a clear effect of intensity of land-use as the forest soil clustered closer to pasture than to the arable soil. The most abundant fungal phyla in the studied soils were Ascomycota, Basidiomycota, and Mucoromycota. Deforestation more than halved the relative abundance of Basidiomycota; mostly Agaricomycetes, such as *Lactarius* and *Inocybe*. The relative abundance of Glomeromycota decreased in the order pasture > forest > arable soil. Symbiotrophs, especially ectomycorrhizal fungi, were negatively affected by deforestation while pathotrophs, especially animal pathogens, were enriched in the pasture and arable soil. Ectomycorrhizal fungi were more abundant in the forest soil as they are usually associated with conifers. Arbuscular mycorrhizal fungi were more abundant in the pasture than in the arable soil as the higher plant diversity provided more suitable hosts. Changes in fungal communities resulting from land-use change can provide important information for soil management and the assessment of the environmental impact of deforestation and conversion of vulnerable ecosystems such as high-altitude temperate forests.

## Introduction

Soil is a non-renewable resource that is under intense pressure by human activities, such as agriculture and husbandry (Olsson et al., 2019). Soil is a highly complex and heterogeneous matrix that supports species assemblage. It is well known that 1 g soil contains up to 10^9^ bacterial cells of 10^4^ taxa, up to 200 m fungal hyphae, and a wide range of different mites, nematodes, earthworms, and arthropods (Bardgett and van der Putten, 2014). This diversity contributes substantially to the total terrestrial biomass and to the ecosystems functioning. Soil fungi play fundamental roles in nutrient cycling and soil health as organic matter decomposers (Lindahl and Tunlid, 2015; Shah et al., 2016), mediators of plant and other soil organisms nutrition (Schneider et al., 2012; Baldrian, 2017), regulating species composition as pathogens (Chapelle et al., 2016), and by providing mutualistic benefits as symbiotrophs (Baldrian, 2017).

Land-use change is a major threat to biodiversity, including belowground diversity, and the ecosystem services that they provide (Bhattacharya et al., 2016). For instance, the soil organic carbon (C) stocks decreased globally 42% when native forests were converted to arable soil (Guo and Gifford, 2002; Bhattacharya et al., 2016). Although deforestation in temperate regions for agriculture or grazing has been reduced mostly or even reversed, it continues unabated in some parts of the world (Currie and Bergen, 2008; Millar and Stephenson, 2015; Food and Agriculture Organization of the United Nations [FAO], 2020) with dire consequences for soil quality. In high-altitude temperate forests in the central highlands in Mexico, conversion of forest to arable land decreased the soil C stock 85% (Fusaro et al., 2019). High soil C stocks reduces the risk of soil erosion and loss of soil fertility, while it facilitates water retention, improves soil structure, and increases soil nutrient content (Lal, 2004). As a consequence, the productivity of maize crops is low in these original forest soils (Montesillo-Cedillo, 2016) and the costs, due to the loss of ecosystems services, are high. Temperate high mountain forests in the tropics are very vulnerable to climate change. If the global temperatures continue to rise, these islands of high biodiversity and endemism would disappear (Halffter and Morrone, 2017; Mastretta-Yanes et al., 2018).

Land-use change induces a series of changes in the abiotic and biotic characteristics of a forest. For instance, the plant community composition (Celentano et al., 2017), edaphic characteristics (Miller and Jastrow, 2000; Lauber et al., 2008; Celentano et al., 2017), availability of plant nutrients (Zhu and Miller, 2003; Wang et al., 2018; Soltangheisi et al., 2019), and the amount and composition of organic matter (Solomon et al., 2002; Raiesi and Beheshti, 2015) are altered by land-use change. All these characteristics are known to affect the soil fungal communities and a number of studies have documented the changes in soil fungi as a result of land-use change in different biomes (Mueller et al., 2016; Marín et al., 2017; Tian et al., 2017). The response of soil fungal communities to land-use change differs across biomes and land-use intensity (Oehl et al., 2010; de la Cruz-Ortiz et al., 2020), depends on the time elapsed since land-use change (Mueller et al., 2016; Zheng et al., 2020), and on the different components of diversity, i.e., species diversity, community composition, and functional guild composition (Pereira et al., 2014; Purahong et al., 2014; Mueller et al., 2016). For instance, fungal species richness was reduced, fungal community composition altered, and generalist fungi enriched when a tropical forest was converted to pasture (Mueller et al., 2016) while it only affected fungal composition in the semiarid Loess Plateau (Tian et al., 2017). In temperate forests, fungal diversity was more linked with vegetation diversity than in any other biome as the ectomycorrhizal host density had a strong effect on the ectomycorrhizal fungal richness (Tedersoo et al., 2014). However, most of these studies have used classic species diversity measures, such as Shannon entropy (Shannon, 1948) and the Gini-Simpson index (Simpson, 1949), that do not measure diversity but entropy. For instance, the Simpson index measures the probability that two randomly chosen DNA sequences truly belong to different operational taxonomic units (OTUs) (Chao et al., 2014). This makes them prone to misuse and misinterpretation (Jost, 2006; Alberdi and Gilbert, 2019). True diversity, on the other side, is based on a mathematical framework easier to interpret and compare between studies (Jost, 2006). As such, it can be hypothesized that conversion of a high-altitude temperate forest to arable land will decrease the soil fungal diversity and specifically ectomycorrhizal fungi in the long-term. Gómez-Acata et al. (2016) found that soil organic carbon content was reduced by deforestation and conversion to arable land and soil characteristics showed a greater variation. As such, forest soils might harbor more heterogeneous microbial communities and thus a higher beta diversity than an arable soil or a pasture soil.

Microorganisms in soil interact with each other within complex food webs. Changes in abundance, diversity and composition of one or more taxa, functional guild or trophic group may affect another group and ecosystems functioning (Wagg et al., 2014). In a forest ecosystem, ectomycorrhizal fungi participate in the decomposition of organic material and become themselves a C substrate and a source of N for bacteria and other fungi (Brabcová et al., 2016).

True diversity indices, or Hill numbers, integrate the most common indices to measure diversity into a single statistical framework that quantify diversity in units of equivalent numbers or equally abundant species or OTUs (Hill, 1973). The weight assigned to the abundant and rare OTUs can be controled using the parameter *q*, known as the “order of diversity” (Jost, 2006). The larger the *q*-value, the higher the importance attributed to abundant OTUs. Consequently, the interpretation of diversity concept is more intuitive and its measurement unit is always the same (Tuomisto, 2010a; Chao et al., 2014). Hill numbers integrate measurement, but also estimation, partitioning, comparison of diversities, and components such as phylogeny and function (Alberdi and Gilbert, 2019). Phylogenetic diversity incorporates evolutionary or phylogenetic differences among species into diversity analysis. In phylogenetic Hill numbers, the units of diversity are branch segments or lineages as defined by a phylogenetic tree instead of OTUs or species, and incorporates the tree’s branching pattern, the relative branch lengths and the relative abundances of each node/branch (Chao et al., 2010). The unit of measurement or the effective total attribute value is the effective total branch length. Similarly, in diversity partitioning using Hill numbers, beta diversity is an actual diversity value that measures the effective number of equally large and completely distinct subsystems in a system (Tuomisto, 2010a,b; Chao et al., 2014).

In this study, the effect on the soil fungal communities of converting a high-altitude temperate coniferous forest to a pasture or agricultural land for the cultivation of maize (*Zea mays* L.) was studied in the natural protected zone “*Área de Protección de Flora y Fauna Nevado de Toluca*” in Mexico using 18S rRNA metabarcoding. Changes in fungal communities resulting from land-use change can provide important information for soil management and the assessment of the environmental impact of deforestation and land-use change. The soil samples used in this study were the same as those used by Gómez-Acata et al. (2016) to study the effect of land-use change on the bacterial community. As such, the effect of land-use change on the soil fungal communities could be compared with that of bacteria.

## Materials and Methods

### Study Site and Soil Sampling

Soil was collected from a coniferous forest (considered the forest soil), deforested for grazing (considered the pasture soil) or maize cultivation (considered the arable soil) at three different locations (“*Rosa Morada*,” “*Dilatada*,” and “*El Capulin*”) in the natural protected zone “*Área de Protección de Flora y Fauna Nevado de Toluca*” (N 19°06′31.5″ W 99°45′17.7″). The vegetation in the forest includes pine (*Pinus* L.), fir (*Abies* Mill.), oak (*Quercus* L.), and mixed forests. Deforestation occurred approximately 50 years ago (Comisión Nacional para Áreas Naturales protegidas [CONANP], 2016). The grassland vegetation was dominated by grasses of the genera *Andropogon* L., *Aristida* L., *Bouteloua* Lag., *Bromus* Scop., *Deschampsia* P. Beauv., *Hilaria* Kunth., *Muhlenbergia* Schreb., *Stipa* L., *Trachypogon* Nees., and *Trisetum* Pers. The arable soil was cultivated with maize, mostly a monoculture but sometimes in rotation with oats (*Avena sativa* L.). The soil was tilled and a limited amount of inorganic nitrogen fertilizer was applied yearly. The average altitude of the three locations was 2,800 m.a.s.l. with an oceanic subtropical highland climate (Cwb) according to the Köppen classification, and a mean annual temperature of 7.5°C (min −2 and max 15°C). The average annual precipitation was 1,100 mm (min 1,000 and max 1,200 mm).

Soil was sampled on March 13, 2015 from the three types of soil (*k* = 3, forest, pasture or arable) from five 400-m^2^ plots, and each type of soil was sampled at three different locations (“Rosa Morada,” “Dilatada,” and “El Capulin”) (*n* = 15) by augering the 0–15 cm layer 20 times at random with a soil auger (7-cm diameter, Eijkelkamp, Giesbeek, Netherlands). The 20 samples taken from each 400-m^2^ plots were pooled so that 45 soil samples were obtained: five 400-m^2^ plots × three soil types × three locations = 45 soil samples. This field-based replication (*N* = 45) was maintained in the laboratory to avoid pseudo-replication. The soil was taken to the laboratory on ice. A part of each soil sample was used for chemical and physical characterization while the rest for extraction of DNA as described below. The characteristics of the soils can be found in Gómez-Acata et al. (2016).

### Soil DNA Extraction, PCR Amplification and High-Throughput Sequencing

Metagenomic DNA was extracted using the Power Soil DNA Isolation Kit (MO BIO Laboratories, CA, United States) according to the manufacturer’s instructions. Fragments of the small subunit (SSU) rRNAs were amplified with nu-SSU-0817 (5′–TTA GCA TGG AAT AAT RRA ATA GGA–3′) and nu-SSU-1196 (5′–TCT GGA CCT GGT GAG TTT CC–3′) primers for micro-eukaryotes (Rousk et al., 2010). Primers contained the Roche 454 FLX adapters and 10-pb tags. Amplifications were done using the *Taq* DNA polymerase (Invitrogen, Carslbad, CA, United States) in quadruplicate. Amplification conditions included a denaturation step at 95°C for 10 min followed by 30 cycles at 95°C for 40 s, at 55°C for 40 s, at 72°C for 50 s, and a final step at 72°C for 10 min. Amplicon libraries were pooled and purified using the QIAquick PCR Purification Kit (QIAGEN, Germany). Sequencing was done by Macrogen, Inc. (Seoul, South Korea) on a Roche 454 GS-FLX Titanium^TM^ pyrosequencer (Roche, Mannheim, Germany).

#### Sequence Processing and Taxonomic Analysis

Raw sequences were deposited in the NCBI Sequence Read Archive under Bioproject PRJNA253630^1^. The QIIME version 1.9.1 software pipeline was used to analyze the pyrosequencing data (Caporaso et al., 2010). Poor quality reads (QS < 25, containing homopolymers >6, length <200 nt, and containing errors in primers and barcodes) were excluded from further analysis. Noise from the FLX chemistry was eliminated with the *denoise_wrapper.py* function within QIIME. OTUs were determined at 98% similarity with UCLUST and with an open-reference strategy using the SILVA_132_QIIME_release^2^. Best BLASTn matches against the SILVA reference data base with clusters at 99.0% similarity were used to assign taxonomy. Taxonomic information, number of observations and sample metadata were used to generate biological observation matrix (BIOM-table) at different taxonomic levels. Non-fungal OTUs were eliminated before downstream analysis. Samples with less than 1,000 reads were eliminated from the data set so that a total of 41 samples were analyzed. Ecological guilds (functional guild, trophic mode, and mode of growth) were annotated using the BIOM-table at OTU level with FUNGuild (Nguyen et al., 2016). Four hundred thirty-nine OTUs out of a total of 623 were assigned to an ecological guild.

### Statistical Analysis

All statistical analysis were done in R (R Core Team, 2016). True diversity Hill numbers were used to measure alpha, beta and phylogenetic diversity at *q* diversity orders of 0, 1, and 2 which consider all OTUs (*q* = 0), frequent OTUs (*q* = 1) and dominant OTUs (*q* = 2) (Chao et al., 2014). The multiplicative partition of diversity in the MetagenomeDiversity script was used for beta diversity analysis (Ma and Li, 2018). The effective number of completely distinct communities is 1 when the communities are identical and 2 when the communities are completely different from each other, i.e., there are no shared species (Jost, 2007). The representative sequence of each OTU (rep_set) was imported into QIIME (version 2-2020.8) to construct the phylogeny to measure the phylogenetic diversity (Bolyen et al., 2019). The rep_set was aligned with MAFFT (Katoh and Standley, 2013) and a rooted maximum likelihood tree was built using IQ-TREE multicore version 2.0.3 (Minh et al., 2019) with the substitution model TIM2 + R8 which is the best model for our dataset according to the ModelFinder algorithm. Hill numbers for phylogenetic diversity were obtained with the *hillR* package in R (Chao et al., 2014).

The effect of land-use change on diversity measures was determined with a linear mixed effects model (LMEM) using the locality as random factor with the *nlme* R package (Pinheiro et al., 2020). The significance of the effect was calculated with 1000 Monte Carlo simulations using the *pgirmess* package in R. The relationship between diversity measures and edaphic characteristics was determined with LMEM with a random intercept for localities using the *lme4* R package (Bates et al., 2015).

Matrices of OTU-observations, taxonomy, and ecological guilds information were imported into R to test the effect of land-use change on the fungal genera and ecological guilds. The data sets were centered log-ratio (clr) transformed by Monte Carlo samplings from the Dirichlet distribution with the ALDEx2 (ANOVA-Like Differential Expression tool for compositional data) R package (Fernandes et al., 2013). A Kruskal–Wallis test was used to find the genera and ecological guilds that had a different abundancy in the different soils. A Benjamini–Hochberg sequential correction was applied to the obtained *p*-value to avoid inflation of Type-I errors. Distance pairwise matrices were calculated using the Aitchison distance (Gloor et al., 2017). Permuted multivariate analysis of variance (perMANOVA) (*n* = 9,999) were performed on Aitchison distance matrices to determine the effect of land-use change on the community structure. Permutations were restricted to the same locality with the strata argument. An analysis of principal components (PCA) and a constrained analysis of principal coordinates analysis (CAP) with the clr transformed matrices and non-collinear physicochemical properties was done with *vegan* R package version 2.4-4 (Oksanen et al., 2018). BIOENV analysis was done to determine which edaphic characteristics produced the highest correlation with the fungal genus and ecological guilds. The bacterial community’s profiles were obtained from the same soil samples (Gómez-Acata et al., 2016), and used to compare the community structure of bacteria and fungi. Procrustes analysis and Mantel test between the Bray–Curtis similarity matrices of the 18S rRNA and 16S rRNA profiles was done with *vegan*.

## Results

### True Diversity

**FIGURE 1:**
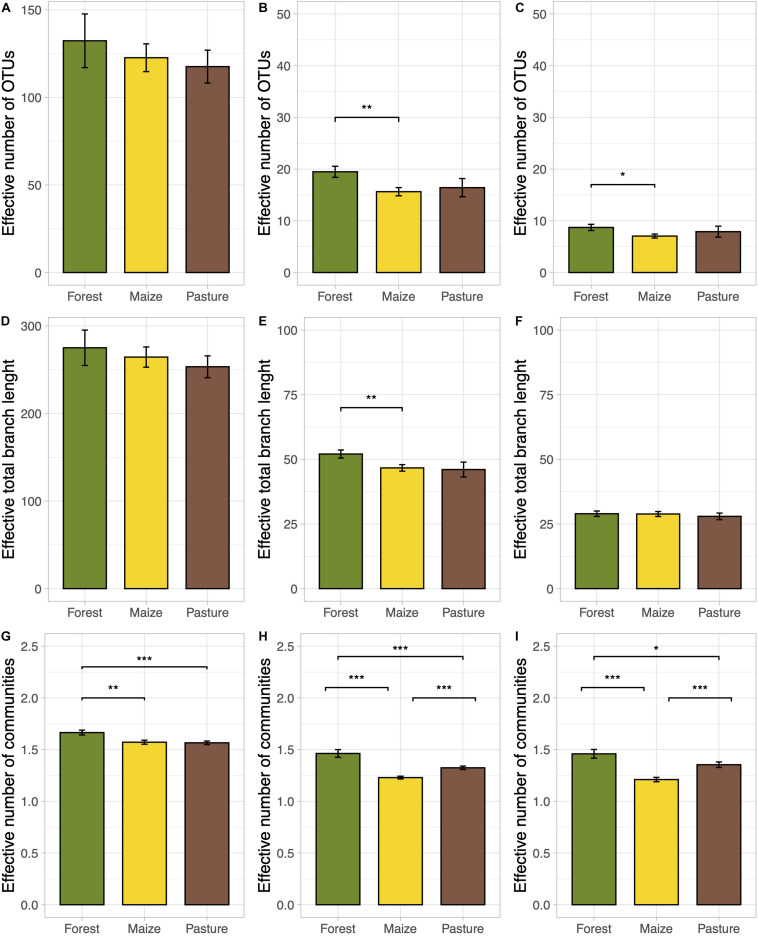
Bar plots of the true diversity parameters of the fungal communities in a coniferous forest soil (Forest), deforested for grazing (Pasture), and in maize (*Z. mays* L.) cultivated deforested soil (Maize) at the “*Área de Protección de Flora y Fauna Nevado de Toluca*” (Mexico). The bar height indicates the mean for each land use system and the error lines indicate the standard error of the mean. Taxonomic alpha diversity **(A–C)**, phylogenetic alpha diversity **(D–F)**, and taxonomic beta diversity **(G–I)** were calculated at diversity orders *q* = 0 **(A,D,G)**, *q* = 1 **(B,E,H)**, *q* = 2 **(C,F,I)**. Linear mixed effects models with 1000 Monte-Carlo permutations and locality as random factor (*p < 0.05, **p < 0.01, ***p < 0.001) was used to test the significant differences.

A total of 132,126 good quality sequences were obtained. Good’s coverage indicated that on average 98% of the fungal diversity was covered per sample. Land-use change did not affect significantly species and phylogenetic richness (*q* = 0; Figures 1A,D). However, the frequent OTUs, species and phylogenetic diversity were significantly lower in the arable than in the forest soil (*p* < 0.05; Figures 1B,E). The phylogenetic diversity of dominant OTUs was not affected by land-use change (Figure 1F). The effective number of dominant OTUs was also significantly lower in arable soil than in forest soil (*p* < 0.05; Figure 1C). The effective number of completely distinct communities was significantly lower in the arable and pasture soil than in forest soil at all *q* diversity levels (*p* < 0.05; Figures 1G–I). The beta diversity was significantly lower in arable than in pasture soil when the typical and dominant OTUs were considered (*p* < 0.05; Figures 1H,I). None of the edaphic characteristics correlated significantly with any components of diversity.

### Fungal Community Structure and Composition

**FIGURE 2:**
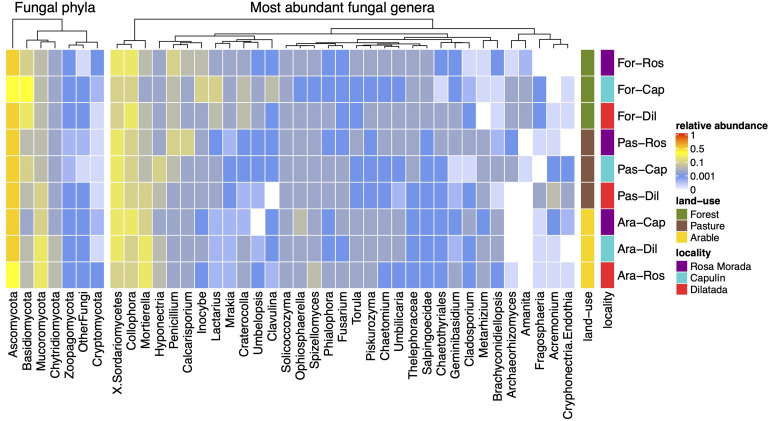
Heat-map of the relative abundance of the fungal phyla and the most abundant fungal genera in a coniferous forest soil (Forest), deforested for grazing (Pasture), and in maize (*Z. mays* L.) cultivated deforested soil (Maize) at the “*Área de Protección de Flora y Fauna Nevado de Toluca*” (Mexico).

The majority of the Fungi belonged to Ascomycota (67.1 ± 13.4%), Basidiomycota (13.1 ± 12.3%), and Mucoromycota (13.1 ± 9.5%) (Figure 2). The relative abundance of Basidiomycota decreased from 25.4 ± 15.0% in the coniferous forest to 8.4 ± 5.3% in the pasture and 6.4 ± 2.5% in the arable soil (χ^2^ = 19.99; df = 2; *p* < 0.0001) while Mucoromycota increased from 7.2 ± 2.9% in forest to 10.1 ± 5.6% in pasture and 21.6 ± 10.7% in arable soil (χ^2^ = 20.76; df = 2; *p* < 0.0001). *Collophora* was the most abundant genus (18.3 ± 9.3%) in all soils.

**FIGURE 3:**
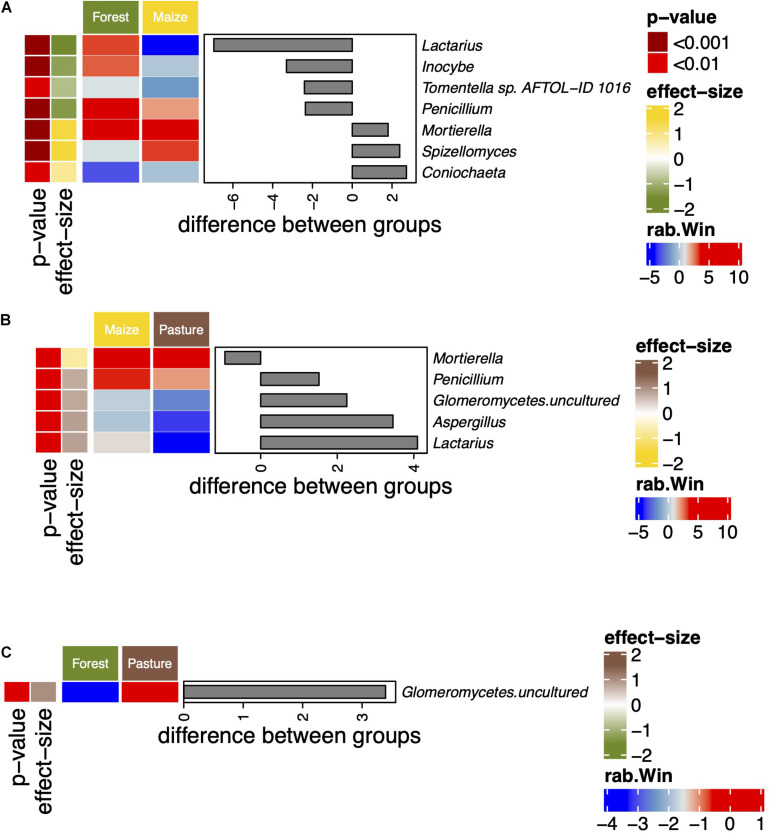
Differentially abundant fungal genera between a coniferous forest soil (Forest), a deforested for grazing (Pasture), and in a maize (*Zea mays* L.) cultivated deforested soil (Maize) at the “*Área de Protección de Flora y Fauna Nevado de Toluca*” (Mexico) as determined by an ANOVA-Like Differential Expression tool for compositional data and Benjamini–Hochberg sequential correction. Comparisons between Forest and Maize **(A)**, Maize and Pasture **(B)**, and Forest and Pasture **(C)** fungal communities.

The abundance of the genera *Lactarius*, *Inocybe*, *Tormentella*, and *Penicillium* decreased significantly when the coniferous forest was converted to arable land while that of *Mortierella*, *Spizellomyces*, and *Coniochaeta* increased (Figure 3). *Penicillium* and *Lactarius* were also more abundant in the pasture than in the arable soil. The relative abundance of uncultured Glomeromycetes was significantly higher in the pasture soil than in the other soils.

**FIGURE 4:**
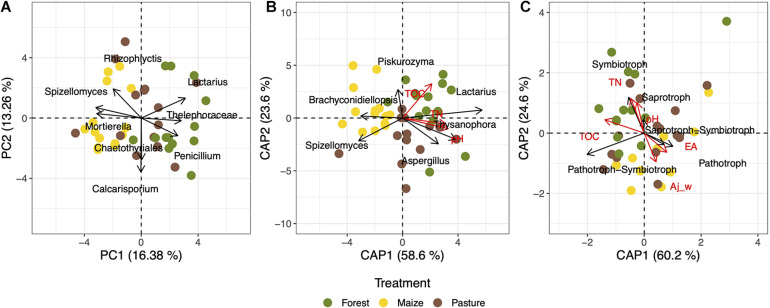
Ordination analyses of the taxonomic composition and trophic groups of fungal communities in a coniferous forest soil (Forest), deforested for grazing (Pasture), and in maize (*Zea mays* L.) cultivated deforested soil (Maize) at the “*Área de Protección de Flora y Fauna Nevado de Toluca*” (Mexico). Principal component analysis (PCA) of the fungal genera with prevalence >20% of soils **(A)**; constrained analysis of principal coordinates analysis (CAP) of the fungal genera with prevalence >20% of soils and total organic carbon (TOC), total nitrogen (TN), and pH **(B)**; and CAP with the trophic fungal groups and electrolytic conductivity (EC), clay content, TOC, TN, and pH **(C)**.

Overall, the structure of the fungal community was affected significantly by land-use (*R*^2^ = 0.062, *p* = 0.002). Fungal communities were not different between localities (*R*^2^ = 0.059, *p* = 0.131) and the effect of the land-use change was independent of the locality (*R*^2^ = 0.098, *p* = 0.397). The PCA of the fungal community structure explained 29.64% when considering the fungal genera that were found in at least 20% of the soil samples (Figure 4A). The ordination analysis showed a clear effect of land-use intensity as fungal communities from forest soil clustered closer to pasture than to arable soil.

### Ecological Guilds of Fungal Communities

**FIGURE 5:**
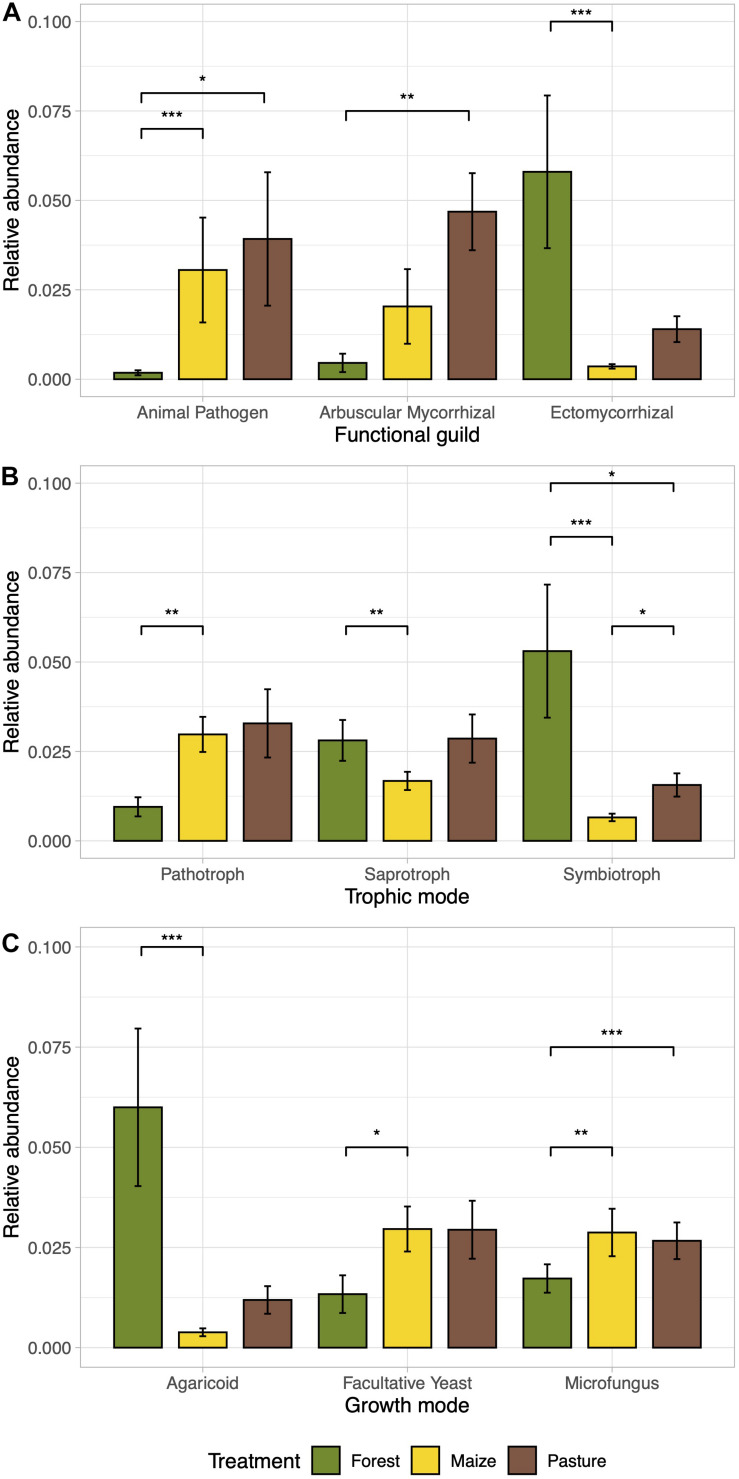
Bar plots of the relative abundance of ecological guilds of the fungal communities differentially abundant between a coniferous forest soil (Forest), a deforested for grazing (Pasture), and a maize (*Zea mays* L.) cultivated deforested soil (Maize) at the “*Área de Protección de Flora y Fauna Nevado de Toluca*” (Mexico). The bar height indicates the mean for each land use system and the error lines indicate the standard error of the mean. Functional guilds **(A)**, trophic mode **(B)**, and growth mode **(C)** as determined by FunGuild software. ANOVA-Like Differential Expression tool for compositional data and Benjamini–Hochberg sequential correction (*p < 0.05, **p < 0.01, ***p < 0.001) was used to test the significant differences.

The relative abundance of ectomycorrhizal fungi decreased significantly in the arable soil (*p* < 0.05; Figure 5A). The arbuscular mycorrhizae were enriched in the pasture soil compared to the other soils, while the relative abundance of animal pathogens increased significantly in the pasture and arable soil compared to the forest soil (*p* < 0.05). Accordingly, symbiotrophs were more abundant in the forest soil while pathotrophs were more abundant in the arable and pasture soil (*p* < 0.05; Figure 5B). Macromycetes were more abundant in the forest soil compared to the other soil and microfungus and facultative yeast in the pasture and arable soil (*p* < 0.05; Figure 5C).

### Fungal Communities and Soil Physicochemical Properties

The CAP analysis considering the abundances of genera and the main explanatory soil physicochemical properties, i.e., pH, total nitrogen (TN), and total organic carbon (TOC), separated clearly the fungal communities of the arable soil from the pasture and forest soils (*F* = 1.66, *p* = 0.006, adjusted *R*^2^ = 0.030) (Figure 4B). The pH alone explained 3% of the fungal composition variance (*p* = 0.031), TN 2.5% (*p* = 0.021), and TOC 1.2% (*p* = 0.101). The pasture and forest soils with a higher pH, TN, and TOC had a higher relative abundance of *Lactarius, Thysanophora* and *Aspergillus* than the arable soil. The model that explained the highest correlation (*r* = 0.144) according the BIOENV analysis contained electrolytic conductivity (EC), TOC, and TN parameters (Table 1).

**TABLE 1:** BIO-ENV Spearman rank coefficients (*r*) of correlations between soil fungal operational taxonomic units (OTUs), genus, ecological guilds, and edaphic soil properties.

	**BIO-ENV factors^*a*^**	***r* coefficient**
Fungal OTUs	EC, TOC, TN	0.144
Fungal genera	Clay, porosity, EC, CEC, TOC, TN, Mg	0.242
Fungal genera with >20% prevalence	Clay, apparent density, TOC, TN, P	0.257
Trophic modes	Silt, EC, TOC	0.286
Functional guilds	Silt, apparent density, porosity, TOC	0.187
Growth mode	Relative density, TOC	0.091

*^*a*^Environmental factors included pH, total organic carbon (TOC), total nitrogen (TN), total phosphorus (P), electrolytic conductivity (EC), apparent density, relative density, porosity, cation exchange capacity (CEC), clay, silt, sand, Mg^++^, Ca^++^, K^+^, and Na^+^ content.*

The CAP analysis with the relative abundance of the trophic modes and all edaphic characteristics (*F* = 6.17, *p* = 0.069, adjusted *R*^2^ = 0.141) separated the fungal communities in the different soils when considering all fungal groups assigned up to the level of genus (Figure 4C). The TOC explained 10% of the variance (*F* = 1.93, *p* = 0.001, adjusted *R*^2^ = 0.101), while the model that give the highest correlation for the trophic modes (*r* = 0.286) contained silt content, EC and TOC (Table 1).

### Comparison Between Soil Bacterial and Fungal Communities

**FIGURE 6:**
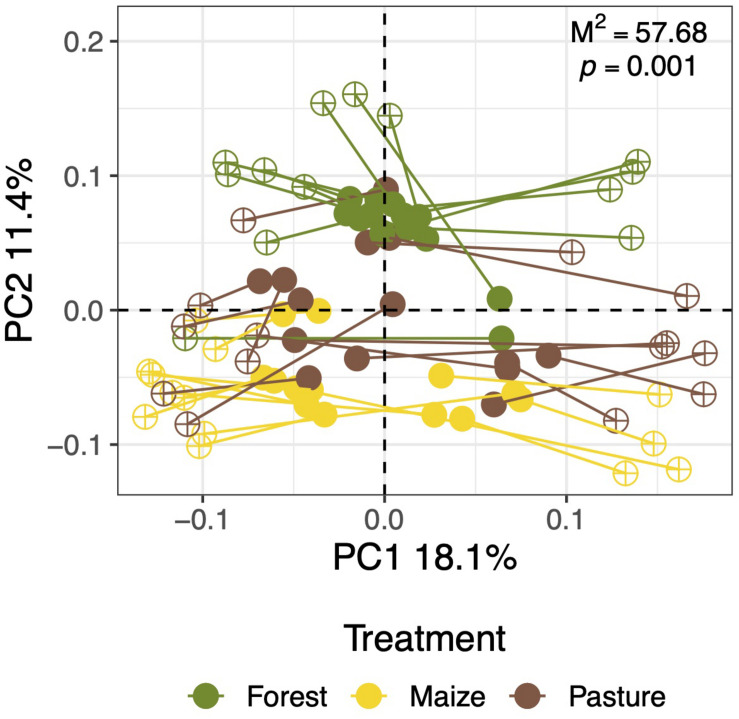
Procrustes analysis of the principal coordinate analysis (PCoA) of Bray-Curtis similarity matrices of the fungal and bacterial communities of the same soil samples collected from a coniferous forest soil (Forest), deforested for grazing (Pasture) and a maize (*Zea mays* L.) cultivated deforested soil (Maize) at the “*Área de Protección de Flora y Fauna Nevado de Toluca*” (Mexico).

The shifts in fungal communities due to the land-use change correlated significantly with those of bacteria determined in the same soil samples (Mantel test; *r* = 0.2068, *p* < 0.05). The changes in beta diversity of bacterial and fungal communities were also significantly correlated using the procrustes analysis (Figure 6). As such, deforestation and cultivation of a high-altitude forest have similar impacts on the composition and taxonomic assemblage of bacterial and fungal communities in soil.

## Discussion

Land-use change is considered a strong driver of biodiversity change (Sala et al., 2000). In this study, we confirmed our hypothesis that the conversion of a temperate forest to arable land significantly decreased alpha, beta, and phylogenetic diversity of soil fungal communities, but only the beta diversity decreased when the forest was converted to pasture. Although the effect of deforestation on species richness and diversity can vary, they decrease mostly when a forest is converted to a cultivated system, such as a plantation, perennial cropland, or cropland (e.g., Balami et al., 2020). In this study, the decremental effect of land-use change on fungal diversity can be mainly attributed to changes in vegetation as none of the tested soil physicochemical properties correlated with changes in the fungal taxonomic and phylogenetic alpha diversity. Although pH decreased in the arable soil compared with the forest soil, pH did not correlate with the decrease in fungal diversity. Species richness of ectomycorrhizal fungi, which was higher in the forest soil in this study, was strongly predicted by the richness of their host plants and pH at larger scales (Rousk et al., 2010; Tedersoo et al., 2014). Experimental studies have shown that acidic soils reduce ectomycorrhizal colonization (Thomson et al., 1996).

The variation in the physicochemical properties and soil fungal communities was smaller in the arable soils compared to the pasture and forest soils (Gómez-Acata et al., 2016). The arable soil from the three locations (*Dilatada*, *Capulin*, and *Rosa Morada*) was cultivated in the same traditional way, i.e., maize monoculture, tillage, and removal of crop residue for fodder or fuel, or it was burned (Arriaga-Cabrera et al., 2009). Similar traditional practices reduced the variation in soil organic carbon stocks in arable soil compared to pasture and forest soils in the high mountain ecosystem of the National Park “*La Malinche*” (Fusaro et al., 2019). The high variation in soil microbial communities in the forest soil might be the result of the high variation in its physicochemical characteristics, e.g., soil organic matter. However, our hypothesis was only partially confirmed as the variation in the edaphic properties did not correlate with the beta diversity of the fungal communities. Mueller et al. (2014) found that changes in the fungal community composition in tropical forest was more closely correlated to plant community composition than to changes in soil properties and Brinkmann et al. (2019) found less dissimilarities between fungal communities at increased intensity in agricultural management. The decline in diversity and the homogenization of fungal communities in the arable soils of the natural protected zone “*Área de Protección de Flora y Fauna Nevado de Toluca*” might be due to the intensive agricultural practices.

Ascomycota and Basidiomycota were the most abundant fungal phyla in the soils studied and are often dominant in soil of different biomes, such as temperate forest (Buée et al., 2009), tropical rainforest (Mueller et al., 2016), arable soil (Klaubauf et al., 2010; Tardy et al., 2015), grassland (Tardy et al., 2015), and arid and semi-arid soils (Tian et al., 2017). The forests soil in this study had a higher abundance of ectomycorrhizal fungi than the other soils, as could be expected as they establish symbiotic relationships with the root system of coniferous plants. Ectomycorrhizal fungi belong mostly to Basidiomycota and to a lesser extent to Ascomycota, which explained why the relative abundance of Basidiomycota was lower in the arable than in the forest soil (Buée et al., 2009).

Glomeromycota establish important mutualisms with plants, i.e., arbuscular mycorrhiza, that allow them to inhabit a wide range of environments and their proportion and diversity is larger in grassland than in forest or arable soils as found in this study (Oehl et al., 2010; Xiang et al., 2014; Belay et al., 2015; Xu et al., 2017). On the one hand, arbuscular mycorrhizae were enriched in the pasture soil compared to the forest soil. Conifers are usually associated with ectomycorrhizal fungi, which also are capable to degrade resistant organic material abundant in a forest soil. On the other hand, the higher plant diversity in grassland provides more niches for arbuscular mycorrhizal fungi than in cultivated soil. Additionally, intensive agricultural practices, such as high fertilizer application rates, pesticide use and plowing, are detrimental for arbuscular mycorrhizal colonization (Hodge, 2014).

Converting a temperate forest to a pasture and arable land also increased the abundance of animal pathogens. In this study, the relative abundance of the fungal genera *Mortierella*, *Phialophora*, *Torula*, or *Acremonium*, which contain pathogenic or opportunistic pathogenic animal species (Davies et al., 2010), was higher in the arable and pasture soils. This increase in animal pathogens in some agroecosystems, often as a result of land-use change, has been associated with the emergence and transmission of infectious diseases (Gottdenker et al., 2014; Mendoza et al., 2020). *Metarhizium* is an entomopathogenic fungus, whose relative abundance decreased in the order arable > pasture > forest. This can be explained by the large increase in the number of insects, such as grasshoppers (*Sphenarium purpurascens* Charpentier), in ecosystems with a limited plant diversity, such as monoculture of maize (Bullen, 1966). Although the relative abundance of plant pathogens did not increase significantly as a result of land-use change, the plant pathogen *Coniochaeta* (described by Damm et al., 2010) was enriched in the arable soil. Similarly, Marín et al. (2017) reported that the intensive management of Chilean temperate forests increased the abundance of fungal plant pathogens. The authors speculate that reduction in the complexity of the vegetation facilitates the dispersion of pathogens.

Fungi that can grow as macromycetes are mostly wood decomposers (saprotrophs) and include several ectomycorrhizal species of forest trees (symbiotrophs). The relative abundance of these functional groups of fungi decreased when the forest was converted to pasture or arable land. Total organic C explained 10% of the variation found in the abundance of fungal trophic groups. Saprotrophs and symbiotrophs play significant roles in soil organic matter formation (Wardle and Lindahl, 2014). Thus, land-use change of a high-altitude temperate forest changes the abundance of saprotrophs and symbiotrophs, which might have a great impact on the ecosystem C exchange and sequestration, and this may, in part, explain the great decline in C stocks in this type of ecosystem (Fusaro et al., 2019).

In a previous study, we found that deforestation had also a profound effect on the bacterial community structure (Gómez-Acata et al., 2016). Bacterial and fungal communities in the soil of the high-altitude temperate forest of the “*Área de Protección de Flora y Fauna Nevado de Toluca*” had a similar sensitivity and similar response to land-use change. Shifts in microbial community composition following land-use change have been associated also with changes in functional genes profiles in high-altitude temperate forest (Muñoz-Arenas et al., 2020). Therefore, land-use change might be strongly and negatively affecting soil functions such as the C and N cycle, which are driven largely by bacterial and fungal communities (e.g., Bardgett and van der Putten, 2014).

## Conclusion

Clearing the temperate high-altitude forest in the “*Área de Protección de Flora y Fauna Nevado de Toluca*” for maize cultivation decreased significantly the effective number of fungal OTUs and their total branch length, but not when converted to grassland. Beta diversity decreased in the order forest > pasture > arable soil. However, none of the measured edaphic characteristics correlated with the changes in alpha, beta, and phylogenetic diversity. The ordination analysis showed a clear effect of land-use intensity as forest soils clustered closer to the pasture soils than to the arable soils. The most abundant fungal phyla in the studied soils were Ascomycota, Basidiomycota and Mucoromycota. Conversion of coniferous forests more than halved the abundance of Basidiomycota, mostly Agaricomycetes such as *Lactarius* and *Inocybe*. The number of OTUs belonging to Glomeromycota decreased in the order pasture > forest > arable soil. Symbiotrophs, especially ectomycorrhizal fungi, were negatively affected by deforestation while pathotrophs, especially animal pathogens, were enriched in the pasture and arable soil compared to the forest soil. The total soil organic carbon significantly explained 10% of the variation of fungal trophic groups. Shifts in the soil fungal communities in these soils as a result of land-use change resembled those of the bacterial communities.

## Data Availability Statement

The datasets presented in this study can be found in online repositories. The names of the repository/repositories and accession number(s) can be found below: https://www.ncbi.nlm.nih.gov/, SRP043580; https://www.ncbi.nlm.nih.gov/bioproject/, PRJNA253630.

## Author Contributions

LD and YN-N designed the study. NM-C, LM-A, and YN-N did the laboratory work. SH-P and YN-N did the bioinformatic analyses. AE-T and YN-N did the statistical analysis. YN-N drafted the initial manuscript. All authors commented on the manuscript.

## Conflict of Interest

The authors declare that the research was conducted in the absence of any commercial or financial relationships that could be construed as a potential conflict of interest.

## Footnotes

1 https://www.ncbi.nlm.nih.gov/sra/?term=SRP043580

2 https://www.arb-silva.de/download/archive/qiime/
